# Innovation in alternate mulch with straw and plastic management bolsters yield and water use efficiency in wheat-maize intercropping in arid conditions

**DOI:** 10.1038/s41598-019-42790-x

**Published:** 2019-04-24

**Authors:** Wen Yin, Zhilong Fan, Falong Hu, Aizhong Yu, Cai Zhao, Qiang Chai, Jeffrey A. Coulter

**Affiliations:** 1 Major in Crop Cultivation and Farming System of Gansu Provincial Key Laboratory of Aridland Crop Science, Lanzhou, China; 2 Major in Crop Cultivation and Farming System of College of Agronomy, Gansu Agricultural University, Lanzhou, China; 3 Department of Agronomy & Plant Genetics, University of Minnesota, St. Paul, MN 55108-6026 United States

**Keywords:** Conservation biology, Conservation biology, Agroecology, Agroecology

## Abstract

In arid regions, higher irrigation quota for conventional farming causes substantial conflict between water supply and demand for crop production. Innovations in cropping systems are needed to alleviate this issue. A field experiment was conducted in northwestern China to assess whether straw and plastic managements in wheat/maize intercropping could alleviate these issues. Integrating no tillage with two-year plastic and straw mulching (NTMI2) improved grain yields by 13.8–17.1%, compared to conventional tillage without straw residue and annual new plastic mulching (CTI). The NTMI2 treatment reduced soil evaporation by 9.0–17.3% and the proportion of evaporation to evapotranspiration (E/ET) by 8.6–17.5%, compared to CTI. The NTMI2 treatment weakened wheat competition of soil moisture from maize strip during wheat growth period, and enhanced wheat compensation of soil moisture for maize growth after wheat harvest, compared with CTI. Thus, soil water movement potential of NTMI2 was lowest during wheat growth period, but it was highest during maize-independent growth stage after wheat harvest. The NTMI2 treatment increased evapotranspiration before maize silking, decreased from maize silking to early-filling stage, and increased after the early-filling stage of maize, this effectively coordinated water demand contradiction of intercrops at early and late stages. The NTMI2 treatment improved WUE by 12.4–17.2%, compared with CTI. The improved crop yields and WUE was attributed to the coordinated water competition and compensation, and the decreased soil evaporation and E/ET.

## Introduction

In densely populated countries, such as Indonesia, India, and China, most of the rural population lives on self-sufficient and small-scale family farms that produce the majority of the country’s grain^1^. To meet the demand of a growing population, grain production must be greatly increased while optimizing the utilization of the limited resources. Small-scale family farms are threatened by deterioration and shortage of natural resources^2^, availability of water resources to compete with urbanization or other sectors^3^, and soil degradation due to unsustainable agronomic practices^4^. Thus, small-scale family farms must adopt innovative cropping patterns that are more productive use resources more efficiently, and have a lighter ecological footprint^5^.

A prominent issue threatening the sustainability of crop production is water use, especially in areas such as northwestern China where available freshwater is 25% less than the internationally-recognized threshold for water shortages^6^. Here, average precipitation over many years is less than 160 mm but annual potential soil evaporation exceeds 2000 mm, causing crop production to relay on irrigation^7^. In the main grain producing areas of northwestern China such as the Hexi oasis irrigation region, the quantity of water available for crop production has seriously declined due to overexploitation of groundwater and uneven distribution across regions, thereby threatening the productivity of current farming systems^8^.

Maize and wheat are the two major food crops planted in arid areas of northwestern China^9^. Both are usually produced with strip intercropping, which allows two crops to be grown within a single season in the same field^10^. This intercropping pattern has been shown to boost crop yields^11^, enhance resource use efficiency^12^, and produce greater economic benefits^13^, compared to the corresponding monoculture crops. However, high yields with this system have been attributed to the large amount of water supplied during the growing season compared to monocropping^14^, creating conflict between increased yields through use of strip intercropping and reduced water consumption for agricultural production. Improvements to this system are needed to concurrently achieve high yields while reducing or optimizing utilization of the limited water resources. In a region with annual soil evaporation greater than 20 times precipitation, how can soil water be best captured with minimized soil evaporation?

Some innovative water-saving measures, such as plastic and straw^7^, regulated deficit irrigation^15,16^, and the implement of policies and bylaws in water management^8^, have been used to promote effective use of water in agriculture. Although high yields with strip intercropping have been attributed to the large amount of water supplied, it is also one of the most effective methods to improve crop water use efficiency. The intercropping combined with regulated deficit irrigation^16^ or straw and plastic mulching^7,17^, can improve water use efficiency in semiarid regions.

However, there is lack of the mechanism responsible for improved water use efficiency in intercropping system. Previous researches have shown that high water use efficiency is because of the increased total yields per unit of water supply^18,19^. When two contrasting crops are cultivated together in alternate strips, aboveground interspecies interactions are beneficial to improve the stereo-structure availability in light capture between the intercrops^20,21^ and enhance photosynthetically active radiation transmittance^22^. Moreover, underground interspecific competition and facilitation may occur simultaneously when two crops are cultivated together in arid regions with high soil water evaporation^23^. It is likely that underground interspecific interactions lead to competition and compensation use of soil water in both intercrop strips, so soil water movement in the intercrops’ rooting zones may also occur^18,24^. However, there is a lack of quantitative researches regarding the magnitude of soil water that may be competed for both two intercrop strips during their co-growth period and compensated for after early-maturing crop harvest. It is unknown whether the two intercrops compete for soil moisture and whether one intercrop may provide a compensation effect to the other intercrop. Current research is limited in soil water competition and compensation under highly efficient soil water-regulating agronomic measures, such as straw and plastic managements. Therefore, it is of great significance to elucidate the mechanism of high yield and efficient utilization of water in intercropping.

With the aforementioned issues in mind, we set out to design an innovative cropping system integrating two key measures - (i) crop intensification via strip intercropping, and (ii) water harvesting via water conservation approaches utilizing straw residue in wheat strips and plastic managements in maize strips. The objectives of this research were to determine the effect of the straw and plastic managements on (a) soil evaporation and water consumption characteristics, (b) grain yield and water use efficiency, and (c) the magnitude of soil water competition and compensation in the two intercrop strips for wheat/maize strip intercropping in an oasis region. The central hypothesis of the study is that straw and plastic managements improve crop yields and water use efficiency of intercropping and that this is related to reduced soil evaporation, enhanced effectiveness of water use, and optimizing the relationship between water competition and complementary utilization of the intercropped strips.

## Results

### Effects of straw and plastic management on crop yields of intercropping systems

Wheat/maize strip intercropping produced significantly greater total grain yield than the monocultures across the three years studied (Table 1). Averaged across years and treatments, intercropping systems produced a total grain yield of 16.4 Mg ha^−1^, which was 11.7–14.5% greater than that of monoculture maize treatments and 1.2- to 1.4-fold greater than that of the monoculture wheat treatments.

**Table 1:** Total grain yield of wheat and maize by cropping pattern and treatment in 2014–2016.

Cropping pattern	Treatment	Year		
		2014	2015	2016
		Mg ha^−1^		
Monoculture wheat	NTSW	7.37de^a^	7.08 cd	7.89c
	NTMW	7.62d	7.20c	8.04c
	TSW	6.96e	6.69d	7.49d
	CTW	6.28 f	6.07e	6.31e
Monoculture maize	NTM2	14.32c	14.24b	14.41b
	CTM	14.89bc	14.52b	14.62b
Wheat-maize intercropping	NTSI2	16.82a	16.49a	17.23a
	NTMI2	16.90a	16.63a	17.49a
	TSI	16.97a	16.50a	16.86a
	CTI	14.86bc	14.61b	14.93b

^a^Within a column for a given year, means followed by different lowercase letters are significantly different at *P* < 0.05.

No-tillage with straw returned to the field significantly boosted grain yield of monoculture wheat (Table 1). Grain yield of the no tillage with straw standing (i.e., NTSW), no tillage with straw mulching (i.e., NTMW), and tillage with straw incorporation (i.e., TSW) treatments was improved by 16.6–24.9%, 18.6–27.3%, and10.2–18.7%, respectively, compared to the conventional tillage treatment (i.e., CTW); especially for the NTMW treatment which produced 7.2–9.5% greater grain yield than the TSW treatment. No tillage with two-year plastic mulching had no significant effect on grain yield of monoculture maize in comparison to conventional tillage with new plastic film mulch applied annually. However, no tillage with straw residue in wheat strips and two-year plastic film mulching in maize strips boosted total grain yield of intercropped wheat and maize. Compared to the conventional intercropping treatment (i.e., CTI), No tillage with wheat straw standing in wheat strips combined with two-year plastic mulching in maize strips (i.e., NTSI2), no tillage with wheat straw mulching in wheat strips combined with two-year plastic mulching in maize strips (i.e., NTMI2), and tillage with straw was incorporated in wheat strips and annual new plastic mulching in maize strips (TSI) increased total grain yields by 13.7–16.5%, 16.0–20.2%, and 16.7–18.3%, respectively.

### Soil evaporation

#### Soil evaporation of different cropping systems

**Figure 1:**
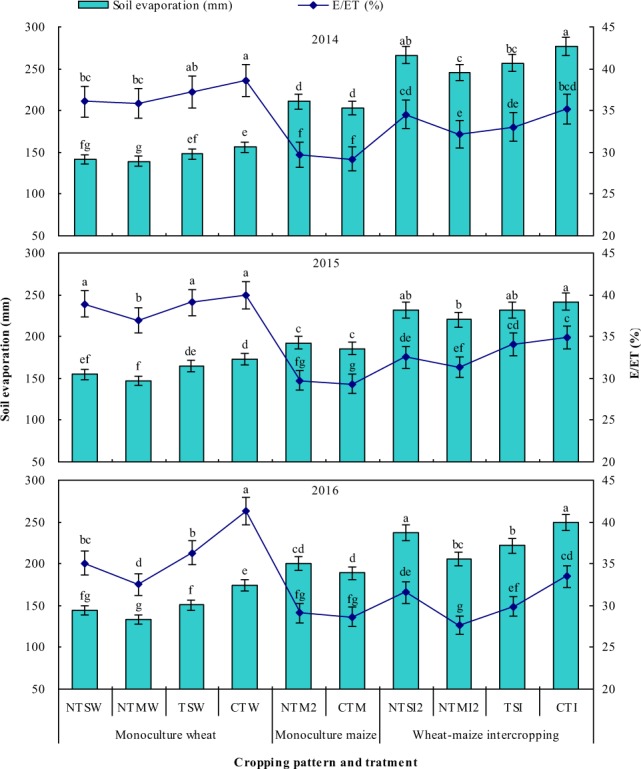
Soil evaporation and the ratio of soil evaporation to evapotranspiration (E/ET) of various treatments under different cropping patterns in 2014–2016. Within a year, different letters indicate significant differences (P < 0.05) among treatments. Error bars are standard errors.

Strip intercropping significantly increased soil evaporation by 44.8–78.5% (higher 72–115 mm) and 17.6–26.2% (34–54 mm) compared to monoculture wheat and maize, respectively (Fig. 1). Straw retention significantly reduced soil evaporation of monoculture wheat. The NTSW, NTMW, and TSW treatments reduced soil evaporation by 9.3–17.4%, 10.8–23.3%, and4.3–13.4%, compared to the CTW treatment, respectively, and soil evaporation of the NTSW and NTMWtreatments was 4.0–5.8% and 5.6–11.4% less than that of the TSW treatment, respectively. Within monoculture maize, soil evaporation was not affected by plastic film mulching approach. However, NTMI2 treatmentsignificantly reduced total soil evaporation by 9.0–17.0% compared to the CTI treatment.

#### Effects of straw and plastic management on soil evaporation of intercropping strips

**Figure 2:**
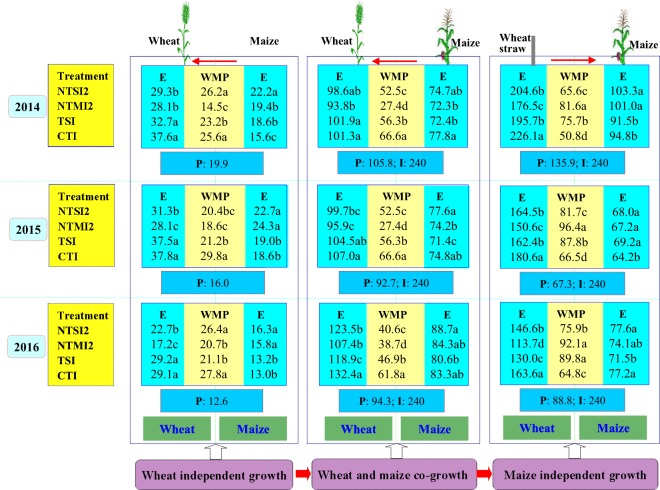
Soil evaporation (E, mm) and soil water movement potential (WMP, mm) of the intercropped wheat and maize strips, along with precipitation (P, mm) and irrigation (I, mm), by crop growing period in 2014–2016. The WMP in the intercropping systems is defined as one-half of the high soil water storage (SWS, mm) minus the low SWS of crop strips for the 0–120 cm soil depth and includes E from the two crop strips, the arrow to the left indicates that soil water has a tendency of moving from maize to wheat strips, the arrow to the right indicates that soil water has a tendency of moving from wheat to maize strips. Within a column for a given year, means followed by different lowercase letters are significantly different at *P* < 0.05.

The NTMI2 treatment inhibited the soil evaporation significantly compared to the CTI (Fig. 2). In 2014–2016, soil evaporation with the NTMI2 treatment was reduced by 7.1–21.6%, 6.4–11.1%, and 11.0–22.0% compared to the control during the wheat-independent growth period, the two crops co-growth period, and the maize-independent period, respectively. These results show that no tillage, in combination with straw mulching the soil surface in wheat strips and two-year plastic mulching in maize strips, was an effective measure to reduce soil evaporation in the intercropping.

During the wheat-independent growth period, soil evaporation in the wheat strips was 32.0–39.3%,8.9–44.8%, 75.8–121.2%, and 103.2–141.0% higher than that in the maize strips for the NTSI2, NTMI2, TSI and CTI, respectively (Fig. 2). Compared to the CTI, soil evaporation in wheat strips of the NTSI2 and NTMI2 was 17.2–22.1% and 25.3–40.9% less, respectively; however, in maize strips it was 22.0–42.3% and 21.5–30.6% greater, respectively.

During wheat-maize co-growth, soil evaporation in wheat strips was 28.5–39.2%, 27.4–29.7%, 40.7–47.5%, and 30.2–58.9% higher than that in maize strips for NTSI2, NTMI2, TSI, and CTI, respectively (Fig. 2). Compared to the CTI, soil evaporation in wheat strips of NTSI2 and NTMI2 was 6.7–7.0% and 7.4–18.9% less, respectively; however, it was not significantly different in maize strips.

During the maize-independent growth period, soil evaporation in wheat strips was 88.9–141.9%, 53.4–124.1%, 81.8–134.7%, and 111.9–181.3% higher than that in maize strips for the NTSI2, NTMI2, TSI and CTI treatments, respectively (Fig. 2). Compared to the CTI treatment, soil evaporation in wheat strips of the NTSI2, NTMI2 and TSI treatments was 8.9–10.4%, 16.6–30.5%, and 10.1–20.5% less, respectively; however, in maize strips it was 5.9–9.0% and 4.7–6.5% greater, respectively. These results show that reducing soil evaporation from wheat strips is important in wheat-maize intercropping, especially after wheat harvest.

### Water competition and compensation between intercropping strips

#### Soil water potential in the 0 to 120 cm soil profile of different cropping systems

**Figure 3:**
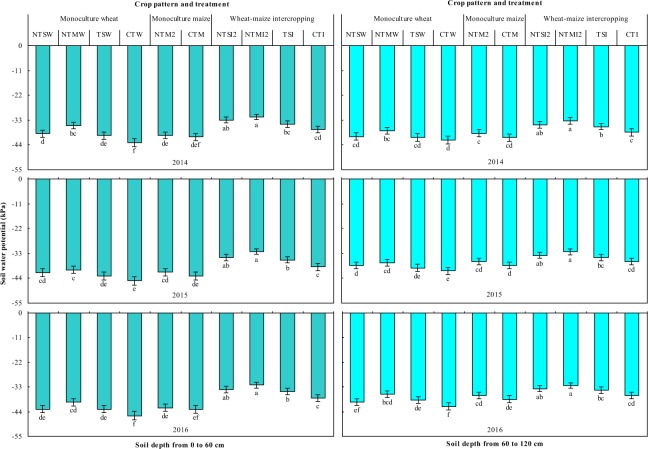
Soil water potential in the 0–60 cm and 60–120 cm soil depth as affected by cropping system in 2014 to 2016. Within a year, different letters indicate significant differences (*P* < 0.05) among treatments. Error bars are standard errors.

Strip intercropping maintained high soil water potential of 0–60 cm and 60–120 cm soil profile in comparison with monocultures, and strip intercropping integrated with straw retention and plastic further improved soil water potential (Fig. 3). Intercropping had 13.2–18.6% and 14.9–18.2% in the 0–60 cm and 11.2–12.1% and 8.2–10.3% in 60 to 120 cm soil profile, greater soil water potential than monoculture wheat and maize treatments, respectively. NTSI2 and NTMI2 increased soil water potential by 22.9–25.5% and 26.7–29.6% compared to CTW, respectively, by 18.3–20.5% and 21.8–25.2% in comparison to CTM, respectively, and by 10.2 to 11.0% and 14.8 to 17.5%, compared to CTI, respectively, in 0 to 60 cm soil profile. From 60 to 120 cm soil profile, NTSI2 and NTMI2 increased soil water potential by 16.3–19.0% and 20.4–22.3% compared to CTW, and by 11.5–14.0% and 15.7–18.3% compared to CTM, and by 7.5–8.6% and 11.9–13.2% compared to CTI, respectively.

#### Effects of straw and plastic management on soil water potential of intercropping strips

**Figure 4:**
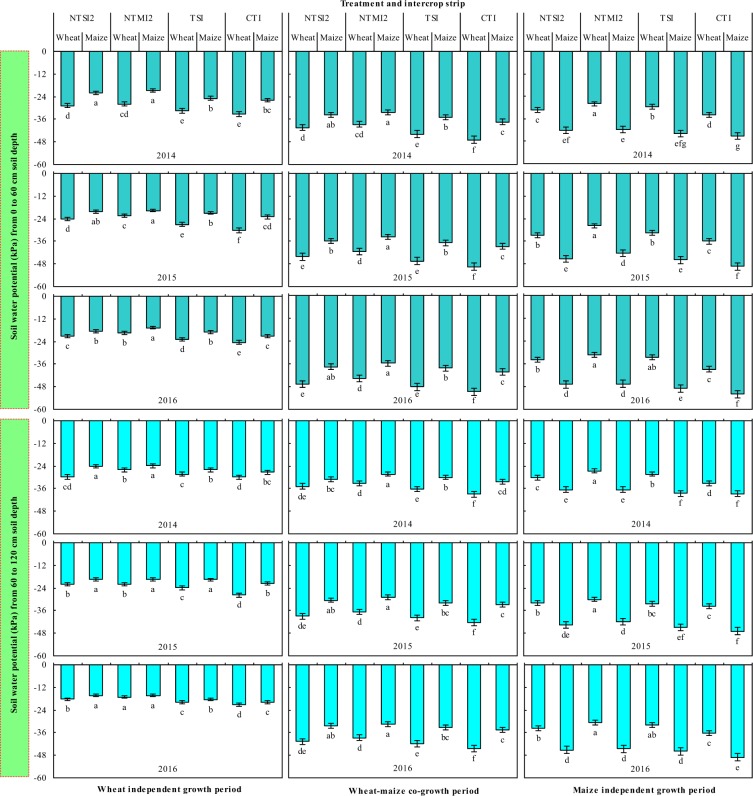
Soil water potential in the 0–60 cm and 60–120 cm soil depth for the intercropped wheat and maize strips in 2014–2016. Within a year and growing period, different letters indicate significant differences (*P* < 0.05) among treatments. Error bars are standard errors.

The difference in the soil water potential of the wheat and maize strips in the intercropping systems is reflected in three growth periods, including the wheat-independent growth period, the intercrops’ co-growth period, and the maize-independent growth period (Fig. 4). During the wheat-independent growth period, soil water potential of maize strips across 0–60 cm soil depth averaged 11.9–23.5%, 12.6–25.4%, 16.7–22.7%, and 12.9–23.7% higher than that of wheat strips in the NTSI2, NTMI2, TSI, and CTI treatments, also, by 10.7–19.0%, 4.3–11.9%, 7.5–17.9%, and 7.4–21.7% across 60–120 cm, respectively. Similarly, during the intercrops’ co-growth period, soil water potential of maize strips of the NTSI2, NTMI2, TSI, and CTI treatments was 17.2–19.5%, 16.6–18.6%, 20.0–20.9%, and 20.3–21.6% higher than that of wheat strips in 0–60 cm, and by 11.0–21.7%, 14.8–21.2%, 17.4–20.6%, and 17.2–22.8%, in 60–120 cm, respectively, and the difference in soil water potential between intercrops strips was reduced with NTMI2. These findings indicate that soil moisture had a tendency to flow from maize to wheat strips, and that NTMI2 reduced the movement potential of soil moisture between intercrops strips compared to CTI, during the wheat growth period.

However, during the maize-independent growth period, soil water potential of wheat strips across 0–60 cm soil depth averaged 25.6–28.1%, 32.8–34.4%, 31.5–33.4%, and 24.8–27.2% higher than that of maize strips in the NTSI2, NTMI2, TSI, and CTI treatments, and by 17.7–27.7%, 27.5–31.5%, 26.2–30.9%, and 13.8–28.2%, respectively, and the difference in soil water potential between intercrops strips was enhanced with NTMI2. These data indicate that soil moisture had a tendency to flow from wheat to maize strips, and that NTMI2 enhanced the movement potential of soil moisture, compared to CTI, after wheat harvest. Overall, the NTMI2 treatment favorably weakened competition for soil water from wheat strips during wheat growth and strengthened soil water compensated to maize strips following wheat harvest, compared with CTI.

During the three periods of crop growth, soil water potential of wheat and maize strips was greater with NTSI2, NTMI2, and TSI than CTI (Fig. 4). Across the four intercropping treatments, the NTMI2 treatment had the greatest soil water potential of wheat and maize strips. These results show that integrated water conservation practices (no-tillage with two-year plastic mulching in maize strips coupled with straw mulching the soil surface in wheat strips, i.e., the NTMI2 treatment) kept a relatively high soil water potential, thereby enhancing the soil water environment for intercropping.

#### Effects of straw and plastic management on soil water movement potential of intercropping strips

Soil water status was monitored in each intercrop strip across the entire growing season, including wheat-independent period, intercrops’ co-growth period, and maize-independent period, to quantify soil water competition and compensation effect of both intercropped strips. Soil water movement potential between wheat and maize strips was expressed as one-half of the difference between the high soil water storage strip and the low soil water storage strip divided by soil evaporation of both strips in intercropping system (Fig. 2). During the wheat-independent growth period, in 2014–2016, soil water moved from maize strip to wheat strip by 20.4–26.4, 14.5–20.7, 21.1–23.2, and 25.6–29.8 mm in the NTSI2, NTMI2, TSI, and CTI treatments, respectively. During the intercrops’ co-growth period, soil water moved from maize strip to wheat strip by 40.6–52.5, 27.4–38.7, 46.9–56.3, and 61.8–69.5 mm in the above four intercropping treatments, respectively. Across the four intercropping treatments, the NTMI2 treatment had the least amount of soil water moved from wheat strip to maize strip, with wheat strip having the lowest level of competition for soil water. Here, we show that intercropping coupled with straw mulching the soil surface and two-year plastic mulching can significantly reduce soil water losses from wheat strips and lower the competition for soil water from the adjacent maize strips during the co-growth period.

During the maize-independent growth period, in 2014–2016, soil water moved from the maize strips to the wheat strips by 65.6–81.7, 81.6–96.4, 75.7–89.8, and 50.8–66.5 mm in the NTSI2, NTMI2, TSI, and CTI treatments, respectively (Fig. 2). This movement of soil moisture from wheat to maize strips after wheat harvest was consistent at all treatments and compensated the growing maize. Across the four intercropping treatments, the NTMI2treatment allowed the greatest amount of the remaining, unused water to be moved from wheat to maize strips, while the CTI treatment allowed the least. This study shows that the treatment with no tillage and straw mulching the soil surface after wheat harvest reduces soil water competition and enhances water compensation for maintaining water balance of two strips in the intercropping system.

#### Effects of straw and plastic management on soil water balance characteristics of intercropping strips

Rainfall and irrigation were the two main water sources in this study. Field evapotranspiration mainly included soil evaporation and crop transpiration, but evapotranspiration of intercrops could not be quantified due to soil water movement potential between intercrop strips. Therefore, it is important to explore the soil water balance between intercrop strips for improving water utilization in intercropping. The treatment on NTMI2 favorably reduced soil evaporation during the entire growth period **(**Fig. 2**)**. Reducing soil evaporation from wheat strips is important in intercropping, especially during the maize-independent growth period. The NTMI2 treatment gave rise to the least amount of soil water moved to the wheat strip from the maize strip, with the wheat strip having the lowest level of competition for soil water during the co-growth period. This treatment allowed the greatest amount of remaining, unused soil water in wheat strips to be moved to maize strips. Therefore, this integrated approach can reduce competition for soil water and enhance water compensation for maintaining soil water balance between two strips in intercropping, and provides an ecological foundation for efficient utilization of soil water in intercropping systems.

#### Evapotranspiration and evapotranspiration modulus coefficient of intercropping at each of growth stage

Strip intercropping treatments had greater total evapotranspiration (ET) than monoculture wheat and maize treatments due to a longer crop growth period (Table 2). The straw and plastic management had no significant effect on total ET during the entire crop growth period in 2014 and 2016; but in 2015 the NTMI2 treatment reduced total ET by 4.7% compared to CTI.

**Table 2:** Evapotranspiration and evapotranspiration modulus coefficient of wheat-maize intercropping at each of growth stage under different straw and plastic managements in 2014–2016.

Year	Treatment^a^	Wheat sowing—maize sowing^b^		Maize sowing—jointing		Maize jointing—pre-heading		Maize pre-heading—silking		Maize silking—early filling		Maize early filling—harvesting		Entire growth
		ET1 (mm)	ET1/ET (%)	ET2 (mm)	ET2/ET (%)	ET3 (mm)	ET3/ET (%)	ET4 (mm)	ET4/ET (%)	ET5 (mm)	ET5/ET (%)	ET6 (mm)	ET6/ET (%)	ET (mm)
2014	NTSI2	37a^b^	4.8a	132b	17.1b	125a	16.2a	138a	17.8a	60a	7.7a	280c	36.3c	772a
	NTMI2	37a	4.4b	132b	17.2b	124a	16.3a	140a	18.3a	59a	7.7a	276c	36.1c	764a
	TSI	33b	4.3b	138a	17.8a	122a	15.7a	141a	18.2a	50b	6.5b	292b	37.7b	776a
	CTI	30c	3.9c	135ab	17.1b	114b	14.5b	144a	18.3a	48b	6.2b	315a	40.1a	787a
2015	NTSI2	40a	5.6a	166a	23.3ab	116b	16.3b	116a	16.3a	58c	8.1c	217a	30.4a	712a
	NTMI2	40a	5.8a	159b	22.6b	124a	17.6a	112a	16.0ab	54d	7.8d	212ab	30.2a	702ab
	TSI	36b	5.3b	162ab	23.9a	123a	18.0a	105b	15.5b	62b	9.1b	192c	28.2b	680b
	CTI	33c	4.8c	163ab	23.6a	121ab	17.4a	102b	14.7c	67a	9.7a	207b	29.9a	693ab
2016	NTSI2	48a	6.4a	176a	23.4ab	108b	14.3b	136a	18.1a	55c	7.3c	230ab	30.5ab	753a
	NTMI2	47ab	6.4a	173a	23.2b	111ab	14.9ab	134a	17.9a	48d	6.4d	234a	31.3a	747a
	TSI	45b	6.0b	178a	24.0a	114a	15.4a	128b	17.2b	59b	7.9b	218c	29.4c	742a
	CTI	41c	5.5c	179a	24.1a	112ab	15.0ab	127b	17.1b	64a	8.6a	221bc	29.7bc	745a

^a^The sampling dates were 18 March, 22 April, 27 May, 19 June, 23 July, 6 August, and 1 October in 2014, 28 March, 23 April, 30 May, 21 June, 27 July, 9 August, and 30 September in 2015, and 29 March, 19 April, 28 May, 26 June, 20 July, 8 August, and 20 September in 2016.

^b^Within a column for a given year, means followed by different lowercase letters are significantly different at *P* < 0.05.

In 2014, there was no consistent trend on the ET and evapotranspiration modulus coefficient (EC) among intercropping systems and was likely because cumulative precipitation during the growing season (262 mm) was 90 cm greater than long-term (1960–2015) average, at entire growth period (Table 2). In 2015 and 2016 during the wheat-independent growth period, compared to CTI, NTSI2 and NTMI2 increased ET by 16.2–22.3% and 10.2–22.1%, and increased EC by 14.9–24.7% and 13.5–20.5%, respectively. From maize sowing to the pre-heading stage, straw and plastic management had no significant effect on ET and EC of four intercropping treatments. From the pre-heading to early-filling stages in maize, the NTSI2 and NTMI2 treatments increased ET by 6.8–14.0% and 4.9–10.5%, and increased EC by 4.7–6.4% and 4.5–9.1%, respectively, compared to CTI. From maize silking to the early-filling stage, the NTSI2 and NTMI2 treatments decreased ET by 13.6–14.2% and 11.2–24.8%, and decreased EC by 14.5–16.5% and 20.2–25.1%, respectively, in comparison to CTI. However, from maize filling to harvesting stage after wheat harvest, NTSI2 and NTMI2 had 3.8–4.6% and 3.5–5.6% greater ET than CTI, respectively; and greater ET by 5.3–12.9% and 7.2–10.6%, greater EC by 3.7–7.8%, 6.4–7.2%, than TSI, respectively.

When compared to the CTI treatment, evapotranspiration with the NTSI2 and NTMI2 treatments was increased until maize silking, decreased from maize silking to the early-filling stage, and increased after the early-filling stage of maize. This effectively coordinated water demand contradiction of intercrops at early and late stages, and a greater extent with the NTMI2 treatment.

### E/ET ratio

#### E/ET ratio of different cropping systems

Strip intercropping significantly reduced the ratio of soil evaporation to evapotranspiration (E/ET) by 8.7–15.6%, compared to monoculture wheat, but improved the E/ET ratio by 5.7–12.6%, compared to monoculture maize, during the entire crop growth period (Fig. 1). During the entire crop growth period, NTMW reduced the E/ET ratio of monoculture wheat by 6.9–21.3% compared to CTW; similarly, NTMI2 significantly reduced the E/ET ratio of intercropping by 8.6–17.5% compared to CTI.

#### E/ET ratio of intercropping systems at various growth stages

During the wheat-independent growth period of the intercropping treatments, the two no-tillage treatments (NTSI2 and NTMI2) significantly reduced the E/ET by 20.3–20.9% and 19.1–31.9%, compared to CTI, respectively, and TSI reduced the E/ET ratio by 6.8–12.1% (Table 3). Meanwhile, NTSI2 and NTMI2 reduced the E/ET ratio by 10.1–14.5% and 8.0–26.9% compared to TSI, respectively. However, from maize sowing to jointing stage, the E/ET ratio of NTSI2 and NTMI2 was increased by 9.2–14.8% and 7.2–10.9% compared to CTI, and was increased by 9.1–21.1% and 10.3–15.1% compared to TSI, respectively. From maize jointing to silking stage, NTSI2 and NTMI2 reduced the E/ET ratio by 10.1–22.6% and 4.4–29.3% compared to CTI, respectively. During the maize filling stage in 2015 and 2016, the E/ET ratio of NTSI2 and NTMI2 was reduced by 5.9–7.0% and 11.6–25.2% compared to CTI, respectively, and the E/ET ratio of NTMI2 was reduced by 12.8–14.7% compared to TSI.

**Table 3:** The ratio of soil evaporation to evapotranspiration (E/ET) of wheat-maize intercropping at each of growth stage under different straw and plastic managements in 2014–2016.

Year	Treatment	Wheat sowing—maize sowing^a^	Maize sowing—jointing	Maize jointing—pre-heading	Maize pre-heading—silking	Maize silking—early filling	Maize early filling—harvesting
%							
2014	NTSI2	69.4c^b^	26.7a	21.2b	18.0b	53.3a	41.6a
	NTMI2	71.0c	26.4a	20.6b	16.2c	42.8c	38.1b
	TSI	77.2b	22.9b	24.0a	18.7b	50.4b	36.6c
	CTI	87.8a	23.8b	25.1a	20.0a	50.9b	37.8bc
2015	NTSI2	67.8c	27.6a	15.4c	21.4c	42.9ab	42.2b
	NTMI2	64.7d	28.0a	13.9d	20.9c	43.6a	40.1b
	TSI	78.6b	25.3b	16.7b	24.9b	41.5c	47.0a
	CTI	85.1a	25.3b	18.0a	27.4a	42.1bc	45.4a
2016	NTSI2	40.6c	28.4a	21.9a	23.9c	51.2b	36.5b
	NTMI2	34.7d	26.5b	18.5c	22.0d	54.3a	29.0d
	TSI	47.5b	23.4d	19.8bc	27.7b	48.0c	33.3c
	CTI	51.0a	24.7c	21.4ab	31.1a	54.0a	38.8a

^a^The sampling dates were 18 March, 22 April, 27 May, 19 June, 23 July, 6 August, and 1 October in 2014, 28 March, 23 April, 30 May, 21 June, 27 July, 9 August, and 30 September in 2015, and 29 March, 19 April, 28 May, 26 June, 20 July, 8 August, and 20 September in 2016.

^b^Within a column for a given year, means followed by different lowercase letters are significantly different at *P* < 0.05.

#### Effects of straw and plastic management on water use efficiency (WUE) of intercropping systems

**Figure 5:**
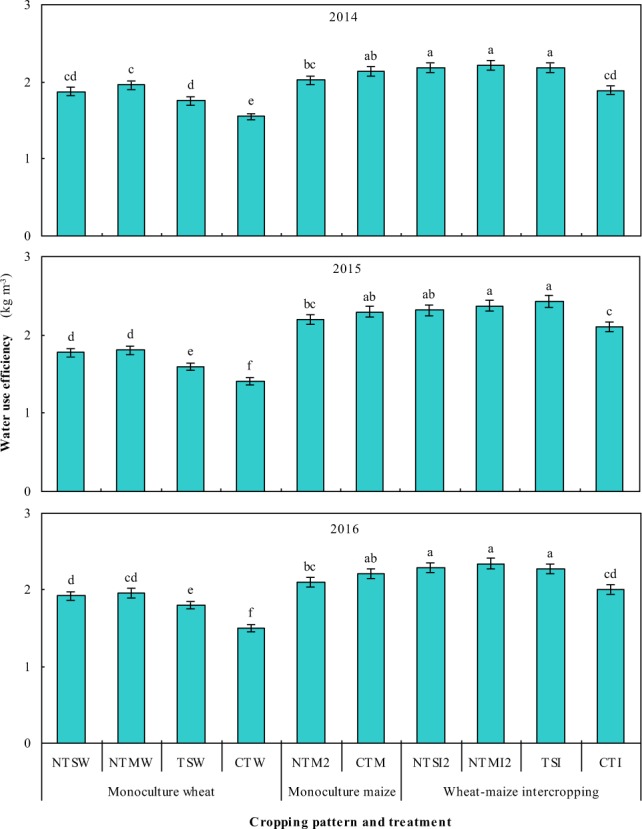
Water use efficiency of various treatments under different cropping patterns in 2014–2016. Within a year, different letters indicate significant differences (P < 0.05) among treatments. Error bars are standard errors.

Strip intercropping significantly enhanced WUE, no tillage with straw retention in wheat strips and two-year plastic mulching in maize strips (i.e., NTSI2 and NTMI2) further improved WUE by 5.5–6.6% and 23.0–44.2% compared to monoculture maize and wheat, respectively. For the monoculture wheat, NTSW, NTMW, and TSW treatments improved WUE by 21.1–28.3%, 26.6–30.6%, and 13.1–20.3% in comparison to CTW during the three study years (Fig. 5). Plastic film mulching patterns did not significantly affect WUE of monoculture maize. Within wheat-maize intercropping, NTSI2, NTMI2, and TSI significantly improved WUE by 9.9–15.3%, 12.4–17.2%, and 13.4–15.8%, respectively, compared to CTI.

## Discussion

The shortage of fresh water is one of the most severe constraints for agricultural production in arid and semiarid areas^8,25^. Climate change may increase the occurrence of extreme weather events in the future^26^, which will undoubtedly have a huge impact on crop productivity^25,27^. It is urgent that effective agronomic measures be adopted to alleviate the challenge. A primary task of agricultural production is to improve effective utilization of water. Reducing ineffective evaporation of soil water is one of the main ways to improve water utilization.

Plastic mulching and straw residue are the two common soil water conservation measures which can enhance water infiltration and increase soil water retention^28,29^. The findings of previous researches showed that no tillage with straw residue and plastic mulching integrated into intercropping can reduce soil evaporation and improve WUE compared with conventional tillage^7,30^. Conventional tillage with annual new plastic mulching is associated with a higher soil temperature in the root zone at the flowering stage of crops, resulting in root and leaf senescence, thus reducing crop yield and water use^31^. However, plastic mulching is imperative for maize production in arid areas which rely on irrigation for crop production^32^. Therefore, this suggests that no tillage with two-year plastic mulching combined with straw residue be applied into crop production, and is evaluated in this study. Of the treatments evaluated, no-tillage in combination with straw mulching the soil in wheat strips and two-year plastic mulching in maize strips (i.e., NTMI2) was the most effective in conserving soil water across the wheat growth period. Soil water under both measures was lost slowly and the available water was maintained for a longer period for crop plants. After wheat harvest, the NTMI2 treatment significantly increased soil water storage by inhibiting soil evaporation compared to CTI. At maize harvest, the effect of NTMI2 on conserving soil water was apparent. Here, we suggest that straw and two-year plastic mulching the soil surface are an indispensable component of wheat/maize strip intercropping. The increased soil water with straw coverage and two-year plastic mulching can partly offset the water deficit in intensified cropping systems. When combined with intercropping, the alternative strip mulching pattern can be an ideal measure for empowering the capacity of soil moisture conservation in arid environments.

The findings of the current research clearly demonstrate that two-year plastic mulching measure integrated into wheat/maize intercropping with straw redisue can decrease soil evaporation, especially wheat strips after wheat harvest. This is because crop residues and plastic mulching the soil surface typically form a barrier against evaporation, thus, maintaining the water storage in the crop root zone^19,33^. Also, straw mulching can reduce soil evaporation via the decreasing of soil temperature^34,35^. In wheat-maize intercropping, the two intercrops have different sensitivity to soil temperature. Wheat is in favor of cooler soil temperature whereas maize is in favor of warmer soil temperature. Optimizing soil temperature to satisfy the requirements for growth of these two intercrops simultaneously has been a challenge. Crop residues left on the soil surface help improve soil roughness, soil surface porosity, and hydraulic conductivity^34^. An added value is that straw mulching reduces soil temperature extremes^36^. Plastic mulch can significantly increase soil temperature^37^, in many cases, the use of plastic can markedly increase soil temperature compared to straw mulch^38,39^. In the present research, two-year plastic mulch is integrated together with wheat straw mulch in wheat-maize intercropping; the innovative technique is that plastic mulching is applied to the maize strips and straw mulch is applied to the wheat strips to balance and optimize soil temperature for the thermophilic maize and the cool-season wheat crops. Solar radiation intercepted on the soil surface can be transferred from maize to wheat strips in the mid to late part of the growing season to provide a heat “buffering effect” between the two crop strips^40^. Consequently, the integration of plastic film with straw mulch can significantly improve microenvironments, thus increasing crop productivity and water utilization. This further confirms the possibility of two-year plastic mulching measure applied to wheat/maize intercropping at a semiarid region. It suggests that the practice of mulching the soil with straw at wheat harvest can be used to glean more precipitation in rainfed farming areas or reduce irrigation amount in irrigated farming areas. Straw residue is a traditional conservation practice, but we found that this traditional technique coupled with two-year plastic mulching in an intensified intercropping system, can be extremely effective for improving yield and water use efficiency in an arid region of water shortage.

Interspecific competition and facilitation are the two main types of interactions in intercropping systems that coexist in mixed systems^13^. Facilitation occurs in most cereal/cereal intercropping systems and occurs as one component species enhances the growth of another species^9^. The competition and compensation of underground nutrients and water is a manifestation of interspecies relationship. In the present study, we determined the soil water competition and compensation for wheat/maize intercropping and found that soil water storage in wheat strips was significantly lower than that in maize strips during the wheat growing season. This was largely due to superior growth of the intercropped wheat compared to the adjacent maize. The cool-season wheat was sown about 30 days earlier than the warm-season maize, providing wheat with a time-advantage for uptake of more available water. Also, it might be possible that greater soil evaporation may have occurred in wheat strips.

In strip intercropping systems, during the early-maturing crop growing period, early-maturing crop roots may expend into the territory of the late-maturing crop, enabling the early-maturity crop to compete for soil water in late-maturing crops strips. However, roots of the late-maturing crop will extend into the early-maturing crop’s rooting regions after the early-maturing crop has been harvested, thereby absorbing some of the remaining but unused soil water, resulting in a water compensation effect to the late-maturing crop^41^. Previous researches have shown that the altered soil water potential is closely related to differences in soil water content, especially in dry soil layers^42,43^. A horizontal driving force is generated between adjacent crop strips due to the differences in soil water content at a given soil depth, thus resulting in a supply of available water for the neighboring crops^44^. Therefore, strip intercropping contributes to an increasing ability to utilize soil water through the gradient difference in soil water storage between intercrop strips for a given soil layer. Asymmetric root systems in intercropping force soil water movement between rooting zones of the intercrops^45,46^. In intercropping, roots of the early-maturing crop are initially deeper than the late-maturing crop, and after harvest of the early-maturing crop, early-maturing crop strips are fallow and roots of the late-maturing crop are dominant in the soil profile. Hence, intercropping improves resource utilization by optimizing crop combination and cultivation measures to improve root morphology. Straw mulching had been shown to effectively conserve soil water and decrease soil temperature and water consumption^47^. In addition, straw mulching has been shown to decrease the depth of crop roots in some cases, leading to weakened root interaction and competition of intercrops during their co-growth period^48^. Therefore, in this study, wheat/maize strip intercropping with wheat straw mulching in wheat strips and two-year plastic mulching in maize strips resulted in the least amount of soil water movement to wheat from maize strip, with the wheat strip having the lowest level of competition for soil water.

After intercropped wheat has been harvested, straw mulching the soil surface can conserve more soil water in wheat strip, and more soil water can then move from wheat to maize strip. This movement compensates for the water requirement for growth of intercropped maize. Intercropping with no-tillage and straw mulching the soil surface in wheat strips and two-year plastic mulching in maize strips allowed the greatest amount of the remaining, unused water to be moved from wheat to maize strips, and conventional tillage treatment allowed the least. Here, we show that no tillage with straw mulching the soil surface in wheat strips combined with two-year plastic mulching in maize strips can weaken soil water competition during the wheat growth period and strengthen water compensation during maize-independent growth after wheat harvest, and thereby maintain water balance between intercrops strips. Hence, these improved practices can result in efficient utilization of water by regulating the water competition and complementary utilization relationship.

## Conclusions

Wheat/maize strip intercropping combined with straw residue and plastic mulching can reduce soil evaporation, decrease the proportion of evaporation to evapotranspiration (E/ET), and improve crop yields and water use efficiency compared to conventional monoculture and intercropping treatments. Strip intercropping combined with straw mulching the soil surface in wheat strips and two-year plastic mulching in maize strips (i.e., NTMI2) was most significant and decreased soil evaporation and the E/ET ratio by 9.0–17.3% and 8.6–17.5% compared to the conventional intercropping treatment (i.e., CTI) across the studying years. During the wheat growth period, wheat strips competed for soil water from the neighboring maize strips of intercropping. After intercropped wheat harvest, maize extracted soil water from wheat strips, which compensated water competition between intercropped strips. The NTMI2 treatment favorably reduced the wheat strip competed soil water from the maize strip and enhanced the wheat strip compensated soil water for the maize strip, compared to CTI. Meanwhile, NTMI2 boosted grain yields by 13.5–19.6% compared to conventional monoculture maize, by 1.7- to 1.8-fold compared to conventional monoculture wheat, and by 13.8–17.1% compared to the conventional intercropping treatment, and improved WUE by 3.2–5.7%, 40.8–64.9%, and 12.4–17.2%, respectively. The 3 year of field experiment showed consistent results that straw and two-year plastic mulching practices applied to wheat/maize intercropping can serve an effective water conservation technique for improving crop yields and WUE in arid irrigation areas.

## Materials and Methods

### Site description

The field experiment was conducted at the Wuwei Agricultural Experimental Station of Gansu Agricultural University near Wuwei City, Gansu Province, China (37°34′N, 102°94′E, 1506 m asl), from 2013–2016. Long-term (1960–2016) annual precipitation is less than 200 mm and annual potential evaporation is greater than 2000 mm. Average annual sunshine duration is 2945 h, solar radiation is 6000 MJ m^−2^, and the frost-free period is 156 d. Heat conditions at this site are suitable for intercropping spring wheat and maize, with an average annual accumulated air temperature ≥ 10 °C of 2985 °C and annual mean air temperature of 7.2 °C. In 2015 and 2016, weather conditions were similar to the long-term average. Precipitation throughout the growth period of the intercrops was 176 mm in 2015 and 196 mm in 2016, whereas in 2014, the growing season precipitation (262 mm) was greater than the long-term average (172 mm). The soil was classified as an Aridisol^49^ with soil bulk density in the 0–110 cm soil depth averaging 1.44 g cm^−3^. The climate and soil of this experimental site is representative of this arid region, where crop production relies on supplementary irrigation.

### Experimental design

The experiment was conducted using a randomized complete block design with three replicates. Treatments were applied to the same plots annually. In 2013, treatments involving different wheat straw management methods combined with two-year plastic film mulching were established in the field prior to the experimental study years 2014, 2015, and 2016. Four wheat straw management approaches were imposed after wheat grain harvest in the late fall of 2013, 2014, and 2015: (i) no tillage with 25–30 cm high wheat straw standing (NTS); (ii) no tillage with 25–30 cm high wheat straw cut 3 cm above the soil surface and evenly distributed over the soil surface (NTM); (iii) conventional tillage (tillage depth of 30–33 cm) using chisel plow with 25–30 cm high wheat straw was incorporated into the soil in late fall (TS); and (iv) conventional tillage without straw residue (CT, all straw cut 3 cm above the soil surface and removed from the plot before tillage in the late fall). These were applied in sole wheat and wheat strips of the wheat/maize intercropping. Two mulching types of colorless plastic (thickness of 0.008 mm) were applied to sole maize and maize strips of the wheat/maize intercropping systems before sowing: (i) no tillage with two-year plastic mulching, and (ii) conventional tillage with annual new plastic mulching. Two types of no tillage with wheat straw retention and two-year plastic mulching produced two no tillage wheat/maize intercropping treatments: (i) no tillage with 25–30 cm high straw standing in wheat strips and two-year plastic mulching in maize strips (NTSI2) and (ii) no tillage with 25–30 cm high straw mulching the soil surface in wheat strips and two-year plastic mulching in maize strips (NTMI2). Conventional tillage with straw incorporation or without straw residue and annual new plastic film mulching produced two conventional tillage wheat/maize intercropping treatments: (i) conventional tillage with 25–30 cm high wheat straw was incorporated into the soil in wheat strips and annual new plastic mulching in maize strips (TSI); and (ii) conventional tillage without straw residue in wheat strips and annual new plastic mulching in maize strips (CTI). The straw retention measures applied to sole wheat produced four treatments: (i) no tillage with 25–30 cm high wheat straw standing (NTSW) and (ii) no tillage with 25–30 cm high wheat straw mulching the soil surface (NTMW); (iii) tillage with 25–30 cm high of wheat straw incorporation (TSW); and (iv) conventional tillage without the wheat straw returned (CTW). The two sole maize treatments were as follows: (i) conventional tillage with annual new plastic mulching (CTM), and (ii) no tillage with two-year plastic mulching (NTM2). This resulted in 10 treatments. Each spring in the conventional tillage treatments, soil in the wheat strips was fertilized, harrowed, leveled, and compacted; then, spring wheat crop was planted with a strip rotary tillage wheat seeder. At the same time, a new plastic was installed on the soil surface in maize strips of the conventional tillage treatments and maize was planted with a manual duckbill punch roller dibbler.

Spring wheat cultivar *Ning-chun* No. 2 and maize cultivar *Xian-yu* No. 335 are widely grown in northwestern China^17^ and were planted in all treatments of this experiment during the three testing years. The sole crop and strip intercropping treatments were sown and harvested on the same date for both wheat and maize. Spring wheat was planted on 21 March, 29 March, 30 March and harvested on 24 July, 28 July, 21 July in 2014–2016, respectively. Maize was planted on 25 April, 24 April, and 20 April and harvested on 1 October, 28 September, and 20 September of the three test years, respectively. The phenological stages of wheat and maize in intercropping system was shown in Table 4. The experimental plot size was 48 m^2^ (10 × 4.8 m). Intercropped wheat and maize were alternated within 160-cm-wide strips and three of these strips constituted one intercropping plot. Each 160-cm-wide intercropped strip consisted of an 80-cm-wide wheat strip with six rows spaced 12 cm apart and an 80-cm-wide maize strip with two rows spaced 40 cm apart. Sole wheat and maize were established at 675 and 8.25 plants m^−2^, respectively, and intercropped wheat and maize were established at 375 and 5.25 plants m^−2^, respectively. Nitrogen and phosphorus were applied using urea and diammonium phosphate. For intercropping treatments, each crop received the same area-based rate of fertilizer as the corresponding sole crop. Nitrogen fertilizer for wheat and maize was applied at 225 and 450 kg ha^−1^, respectively. Phosphorus fertilizer for wheat and maize was applied at 150 and 225 kg P_2_O_5_ ha^−1^, respectively. All N and P fertilizer for wheat and all P fertilizer for maize was broadcast and incorporated prior to sowing. For maize, 30% of N was broadcast and incorporated prior to sowing, 60% was topdressed at the jointing stage, and the remaining 10% was topdressed at the filling stage. All plots received 1200 m^3^ ha^−1^ of irrigation water in the early winter after crops were harvested but before tillage treatments and plastic mulch treatments were applied. Then, all plots received 750, 900, and 750 m^3^ ha^−1^ of irrigation at the seedling, booting, and beginning grain filling stage of monoculture wheat, respectively. Monoculture and intercropped maize received 900, 750, 900, 750, and 750 m^3^ ha^−1^ of irrigation at the jointing, pre-heading, silking, flowering, and filling stage of monoculture maize; additionally, 750 m^3^ ha^−1^ of irrigation was applied to intercropped maize at the seedling stage. Polyvinyl chloride (PVC) pipes (15 cm diameter) were used for plot flooding irrigation. A flow meter was installed to record irrigation amount of each plot.

**Table 4:** The phenological stages of wheat and maize in intercropping system.

Crop growth period	Crop	
	Wheat	Maize
Wheat-independent growth period	Sowing	—
Wheat-maize co-growth period	Seedling stage	Sowing
	Jointing stage	Seedling stage
	Booting stage	Jointing stage
	Heading stage	Post-jointing stage
	Flowering stage	Pre-heading stage
	Filling stage	Heading stage
	Full-ripening stage (harvesting)	Silking stage
Maize-independent growth period	—	Flowering stage
	—	Filling stage
	—	Full-ripening stage (harvesting)

### Measurement and calculation

#### Soil water potential

Soil water potential (kPa) in each plot was measured from the 0–120 cm layer in 30-cm increments using a set of mechanical tensiometers with accuracy of 0.1 Pa (TEN-45, Tuopu, Zhejiang, China), included four types of tensiometers (30, 60, 90, 120-cm), at 5-day intervals during the entire crop growth season. At each measurement, four readings were taken in monocropped plots and in two locations of one wheat and one maize strip of intercropping plots. For each plot, the mean of all readings for a given sampling date was used to represent the plot.

#### Soil evaporation

Micro-lysimeters were used to measure soil evaporation from crop inter-rows from sowing to harvest in each plot as previously used by other researchers^50^. All micro-lysimeters were constructed using PVC tubes with a length of 15 cm, internal diameter of 11 cm, and external diameter of 11.5 cm. The base of the tubes was sealed with waterproof tape. At each measurement, one micro-lysimeter was installed in the central rows of monocropped plots and in two locations of one wheat and one maize strip of intercropping plots. The micro-lysimeter was filled with soil and placed into a larger (internal diameter 12 cm) PVC tube, which was positioned in the plot. Micro-lysimeters were weighed at 18: 00 at intervals of 3 to 5 days, and soil evaporation was calculated from the weight loss of the micro-lysimeters. Weight loss was recorded using a portable electronic balance (1 g = 0.1053 mm of soil evaporation). At each measurement, two readings were taken for each of the wheat and maize strips for the intercropped plot.

#### Evapotranspiration and evapotranspiration modulus coefficient

The approximate evapotranspiration (*ET*, mm) of each plot was calculated using the water balance equation as follows^8^:

1 $$ET={P}_{e}+I+U\,\mbox{--}\,R\,\mbox{--}\,{D}_{w}\,\mbox{--}\,{\rm{\Delta }}S$$

where *P*_*e*_ is effective precipitation (mm), determined by the USDA Natural Resources Conservation Service’ in the late 1990s^51^, *I* is irrigation quota (mm), *U* is upward capillary flow from the root zone (mm), R is runoff (mm), *Dw* is downward drainage out of the root zone (mm), and *△S* is the change in soil water storage in the 0–120 cm layer (mm), from sowing to harvest of crops. Upward and downward flow was measured previously at a nearby field and was found to be negligible in this semiarid region. Runoff was also negligible due to small quantities of precipitation and irrigation that was controlled by raised ridges between plots.

Soil water content (%) before sowing and after harvesting of crops was measured from the 0–30 soil layer in 10-cm increments using the gravimetric method (oven-drying soil samples), and from the 30–120 cm soil layer in 30-cm increments using a neutron probe (NMM 503 DR, CA, USA). At each measurement, one probe was installed in the central rows of monocropped plots, and the probes were installed in the central rows of the wheat and maize strips, the aim is to quantify soil water content in different crop zones.

Volumetric soil water content (cm^3^ cm^−3^) of 0–30 cm was obtained by multiplying gravimetric water content by the corresponding soil bulk density (1.40, 1.42, and 1.47 g cm^−3^, respectively) for the 0–10, 10–20, 20–30 cm soil layers, respectively. Soil bulk density was determined from undisturbed soil cores taken at sowing. Volumetric soil water content was converted to soil water storage (*SWS*, mm) for a given soil layer as follows:

2 $$SWS=\theta v\times h\times 10$$

where *θ*_*v*_ is volumetric soil water content for a specific soil layer (cm^3^ cm^−3^), and *h* is the soil depth increment (cm).

The evapotranspiration modulus coefficient was calculated by ET of different growth stages divided by total ET across the entire growth period.

#### Soil water movement potential

Soil water movement potential (*SWMP*, mm) in the intercropping systems is defined as one-half of the high soil water storage (*SWS*, mm) minus the low SWS of crop strips at 0–120 cm soil depths and includes soil evaporation from the two crop strips. In addition to soil evaporation from the wheat and maize strips, if the late-maturing maize strips have greater soil water storage than the early-maturing wheat strips at a given soil depth, then, there is water competition from wheat strips to maize strips, and water competition is defined as the amount of soil water absorbed by wheat plants from the maize strips during the co-growth period. Alternately, if the early-maturing wheat strips have greater soil water storage than the late-maturing maize strips, then, there is water compensation from wheat strips to maize strips, and water compensation is defined as the amount of soil water absorbed by maize plants from the wheat strips after wheat harvest. Soil water movement potential is calculated as follows:

3 $$SWMP=(SW{S}_{{\rm{high}}}-SW{S}_{{\rm{low}}}-{E}_{W}-{E}_{M})/2$$

where *SWS*_high_ and *SWS*_low_ are high and low soil water storage of intercrop strips, respectively, and *E*_*W*_ and *E*_*M*_ are soil evaporation of intercropped wheat and maize strips, respectively.

#### Grain yield and water use efficiency (WUE)

All plots were harvested artificially when wheat and maize reached physiological maturity, and the grain yields based on air-dried weight were obtained for each crop of the various treatment groups.

WUE was determined using the following equation:

4 $$WUE=GY/ET$$

where *GY* is grain yield (kg ha^−1^) and ET is total evapotranspiration over the whole growing season (mm), calculated from Eq. (1) described above.

### Statistical analysis

The SPSS program (SPSS software, 17.0, IBM Company, USA) was used to conduct analysis of variance. Treatment means were compared using Duncan’s multiple-range test at the 0.05 probability level. Treatment as fixed factor, because the treatment factors were applied in the same experimental plots during the 3 years of research, year and year × treatment as random factors. If the interaction of Treatment and Year was found significant, data were analyzed in separate years.
